# Prediction of maternal quality of life on preterm birth and low birthweight: a longitudinal study

**DOI:** 10.1186/1471-2393-13-124

**Published:** 2013-06-02

**Authors:** Panchalli Wang, Shwu-Ru Liou, Ching-Yu Cheng

**Affiliations:** 1 Department of Obstetrics and Gynecology, Chia-Yi Christian Hospital, 539 Jhongsiao Rd, Chia-yi City 600, Taiwan; 2 Chang Gung University of Science and Technology, 2 Chiapu Rd. West Sec. Putz, Chiayi 613, Taiwan

**Keywords:** Quality of life, Preterm birth, Low birthweight, Pregnancy, Postpartum

## Abstract

**Background:**

Preterm birth is a significant cause of newborn morbidity and mortality and strains society’s healthcare resources due to its long-term effects on the health of the newborn. Prenatal maternal quality of life (QoL) may be related to the occurrence of preterm birth and low birthweight infants. Few studies, however, have investigated maternal QoL, especially throughout the continuum of pregnancy and the immediate postpartum period. Therefore, the purposes of this longitudinal study were to measure the levels of QoL during and immediately after pregnancy in women with uncomplicated pregnancies, investigate the relationships between the dimensions of QoL, and determine whether prenatal QoL can predict preterm birth and low birthweight.

**Methods:**

Using convenience sampling in one hospital in Taiwan, we recruited 198 pregnant women without pregnancy complications after 24 gestational weeks and followed up monthly until one-month postpartum. The Duke Health Profile was used to measure QoL. Data were analyzed using descriptive statistics, the Mann–Whitney *U* test, the Kruskal-Wallis test, generalized estimation equations, Pearson correlations, and hierarchical logistic regression.

**Results:**

Pregnant women did not perceive that they had a high level of QoL. Women at late pregnancy experienced a significant decrease in their level of physical and general health. After childbirth, although the mothers had better physical health, they had poorer social health. Poor QoL at late pregnancy predicted preterm birth. Employment, parity, educational level, and happiness about pregnancy were related to prenatal maternal QoL; employment was a factor related to postpartum maternal QoL.

**Conclusions:**

Early assessment of QoL, including its dimensions, of pregnant women may help us to understand women’s health status. Based on this understanding, healthcare professionals can develop interventions to promote pregnant women’s QoL and to lessen the occurrence of preterm birth and low birthweight infants. Further, an emphasis on the positive aspects of pregnancy may increase maternal QoL.

## Background

It is well known that preterm birth is a significant cause of newborn death. Moreover, the costs of hospitalization and treatments for a premature birth (before 37 gestational weeks) or low birthweight baby (body weight less than 2500 grams) are high [1], and, if the infant survives, the long-term effects on the health of the baby create additional strains on society’s healthcare resources. In 2010, the worldwide preterm birth rate, when combining rates in countries of all developmental levels, excluding Taiwan, was 11.1% [2]. In Taiwan in 2010, the preterm birth rate was 9.3%, and, of these preterm babies, 53.3% were low birthweight babies [3].

Maternal physical, mental, and social health are related to preterm birth or low birthweight infants and include such factors as maternal stress, negative affects, low psychosocial health status, previous or present pregnancy complications, genitourinary infections, multiple fetuses, and lack of perceived social support [4-7]. Thus, overall maternal quality of life (QoL) has become a focus of research that aims to provide a more in-depth understanding of the role of QoL on maternal morbidity and mortality as well as on the incidence of preterm birth.

The QoL of pregnant women affects maternal health as well as fetal and infant health. The majority of previously published studies, however, have focused on specific maternal and/or fetal health issues or complications in pregnancy, and few studies have investigated either overall maternal QoL, including its physical, mental, and social health dimensions, throughout pregnancy and the postpartum period or the effect of QoL on maternal and birth outcomes.

### Overall quality of life

Several instruments that measure QoL including the Short Form-36, Short Form-12, and the World Health Organization Quality of Life Scale, have been used in studies on maternal QoL. All these scales have several dimensions of QoL, for example physical health, psychological health, and social relationships. However, rather than the overall QoL of pregnant women, dimensions of QoL were reported in those studies [8-13]. Through the use of the WHO-5 Well-Being Index in a study of German women, researchers found that 18.6% of those who were between 28 and 35 weeks pregnant and 21.6% of those who were postpartum experienced low well-being, while the rates were 12.8% and 31.3%, respectively, for American women [14]. Using the Maternal Postpartum Quality of Life scale, Hill found that American postpartum women at week 3 had better QoL than women at week 1 [15].

### Maternal physical health

Among the few published studies that have investigated the QoL of pregnant and/or postpartum women, the findings suggest that these women are likely to experience poor physical health [9,16-18]. In a study conducted in Canada, researchers found that pregnant women in their third trimester had lower physical function and vitality and higher role limitation, due to poor physical health and bodily pain, than did non-pregnant women [16]. In a Swedish study, Schytt and Hildingsson found that 20.4% of women at mid-pregnancy (17–19 gestational weeks) perceived having poor physical health; the rate increased to 36.9% at late pregnancy (32–34 gestational weeks) and was 33.7% at one-year postpartum. The researchers also reported that pregnant women who experienced neck/shoulder pain or back pain were more likely to perceive having poor physical health [17]. In an earlier study conducted in the United States, Haas and colleagues found that pregnant women’s physical functioning and vitality decreased from pre-pregnancy to late pregnancy [18]. Australian researchers found that, compared to the general population, pregnant women at 30–32 weeks of gestation perceived having poorer physical health, yet they had better general health [9]. A large study in Turkey found that postpartum mothers had a median high score on a QoL scale and that age, educational level, economic status, method of delivery, and number of children were related to maternal QoL [19].

Studies indicate that mothers’ physical health affects their infants’ health and well-being. For example, poor maternal health has a negative impact on the mothers’ infant care behaviors [20,21], and, as a result, their children experience poor physical health, emotional difficulty, and behavioral problems at three years of age [22].

### Maternal mental health

Postpartum depression has been widely studied and is known to negatively affect women’s QoL status [12]. Few investigations, however, have examined overall maternal mental health and QoL during and after pregnancy. Among this research, a few studies have found that pregnant and/or postpartum women may experience poor mental and emotional health. Although Canadian researchers found that pregnant women perceived that they had about the same level of mental health as non-pregnant women [16], researchers in Australia found that pregnant women at 30–32 weeks of gestation perceived having poorer mental health than did the general population [9]. In a Swedish study, 14.3% of women at mid-pregnancy (17–19 gestational weeks) perceived having poor emotional health; the rate increased to 22.2% in late pregnancy (32–34 gestational weeks) and was 23.9% at one-year postpartum [17].

Poor maternal mental health during pregnancy not only affects pregnant women’s QoL but can also influence their birth outcomes. In the United States, researchers found that pregnant women with low psychosocial health status or negative affects at 22–24 weeks of gestation were more likely to have a preterm birth and low birthweight baby [6].

### Maternal social health

The effect of maternal social health during pregnancy is another under-reported aspect of maternal QoL. According to Larson, social health is an internal response to social stimuli and perceived social support [23]. Findings from the few studies that have examined maternal social health not only indicate that pregnant women are likely to experience negative social health issues but also suggest that poor maternal social health during pregnancy affects birth outcomes. When compared to non-pregnant women [16] or to people in general [9], pregnant women perceived having a lower level of social function. Results from a large retrospective survey conducted in the United States revealed that the number and level of social health issues were related to low birthweight [24]. In other studies, dissatisfaction with social support was related to depression in all trimesters during pregnancy and resulted in somatic symptoms in the third trimester [25] and preterm birth [5].

Despite the effect of maternal QoL on birth outcomes, few studies have examined the relationship of QoL with its dimensions, especially over time [11,15]. Therefore, we conducted a longitudinal study to explore the levels of QoL in women during and after pregnancy, the relationships between dimensions of QoL, and the potentially predictive ability of prenatal maternal QoL for preterm birth and low birthweight. The following research questions guided our study: (a) What are the levels and changes of maternal QoL during and after pregnancy? (b) Do levels of maternal QoL differ by demographic variables? (c) How do dimensions of QoL relate to each other? and (d) Can prenatal QoL predict preterm birth (less than 37 gestational weeks) and low birthweight (less than 2,500 grams)?

## Methods

### Design

This report is part of a larger study that survey maternal health status and birth outcomes. The study was conducted in two stages. In the first stage, we used a cross-sectional design to translate, back-translate, and test psychometric properties of questionnaires if the questionnaires have no Chinese version. In the second stage of the study, we used a prospective longitudinal design to explore levels of QoL in women from pregnancy to the postpartum period. The participants were recruited when they were over 24 gestational weeks, and they were interviewed four separate times during monthly follow-up visits until one-month postpartum.

### Setting

Participants were recruited from a hospital in southern Taiwan. Data were collected from interviews with the participants while they were in the hospital waiting for their prenatal checkups. Data collection occurred from February 2010 to October 2011.

### Sample

The population of the study was pregnant women in the city of Chiayi, Taiwan. Inclusion criteria for participation were women who: (a) were over 17 years old; (b) could read and write Chinese; and (c) were over 24 weeks of gestation without pregnancy complications, including a diagnosis of prenatal depression or anxiety disorder.

The sample size for the pilot study was 130 pregnant women. Using the G*Power statistical power analysis software program [26], with a power of 90% and correlation coefficient between physical, mental, and social health (*r* = −.31 to -.86) found in the pilot study of this current study, the sample size needed for this current study was determined to be 105. Because the study used a longitudinal design, which can often have a high attrition rate of participants, we recruited as many pregnant women as we could during the study period.

Of the 265 pregnant women who were invited to participate in the study, 56 declined, and 209 completed the initial survey. Eleven participants dropped out after completing the second survey, leaving 198 participants who completed all four surveys (as described below). The attrition rate was 6.2%. The women who dropped out of the study and who remained in the study did not significantly differ in any of the demographic variables, which included age, marital status, parity, educational level, employment, whether they were happy about their pregnancy, and whether they had a planned pregnancy.

### Instruments

The Duke Health Profile (DUKE) was used to measure the QoL of the study participants. The DUKE measures physical, mental, social, general, and perceived health. It is a 17-item, three-point measure derived from the Duke-UNC Health Profile (available at http://healthmeasures.mc.duke.edu/). The scores on all subscales of the DUKE are converted to range from 0 to 100, with higher scores indicating better health. The reliability of the scale is supported by Cronbach’s alpha and test-retest correlations. Concurrent and discriminant validity of the scale also is evidenced in the scale development study [27]. For our study, the Chinese version of the DUKE that was provided by the scale developer was used in our study, and the Cronbach’s alpha of the DUKE was tested (alpha = .75) only at initial data collection.

The demographic information was collected through questions that concerned age, educational level, employment, parity, whether the pregnancy was planned, and whether they were happy about the pregnancy. After childbirth, the participants were asked about their baby’s sleep pattern and whether they had someone to take care of them. Their medical records also were reviewed to collect information about the infants.

### Procedure

Participants were recruited via professional referral and personal contacts in the hospital when the women were over 24 weeks of gestation. All participants were asked to complete a set of paper questionnaires administered during four separate time periods: 25–29 gestational weeks (T1); 30–34 gestational weeks (T2); over 34 gestational weeks (T3); and four to six weeks postpartum (T4). Appointments for all four data collection periods were made in accordance with the dates of the participants’ prenatal care checkups at the hospital.

### Ethical considerations

Before beginning the study, the research protocol was approved by the Ditmanson Medical Foundation Chia-Yi Christian Hospital (IRB# 098058) in Taiwan, where the data were collected. In addition to participants’ being informed about the research procedure, they also were informed about confidentiality, privacy, the right to end their participation, and potential benefits and risks. A signed consented form was obtained before data collection. All questionnaires were anonymous, and files that included the participants’ contact information were shredded after all data were collected. Only research-related personnel could access and use the data.

### Data analysis

All collected data were managed and analyzed using SPSS, Version 18.0. Descriptive statistics were used to examine the participants’ demographic information and levels of the measured variables. The Mann–Whitney *U* test and the Kruskal-Wallis test were used to compare differences in measured variables by demographic groups. Pearson correlations and hierarchical logistic regression were used to test the relationships between variables and the predictive ability of variables for newborn preterm birth and low birthweight. Generalized estimation equations (GEE) were used to test changes in measured variables over time. Measured variables were tested for their differences by demographic variables; demographic variables that showed differences on measured variables were controlled for in the GEE analyses.

## Results

### Demographics

The mean age of the participants was 29.74 (*SD* = 4.43, range = 19–42) years, and the mean gestational age at the first data collection time period was 27.34 (191 days, *SD* = 1.08, range = 25–29) weeks. The majority of the participants were primiparas (*n* = 111, 56.1%), and approximately one-third were pregnant with their second child (*n* = 64, 32.3%). Most of the participants were married (*n* = 195, 93.4%) and had planned their pregnancy (*n* = 102, 51.5%). Additionally, 50.0% (*n* = 99) were “very happy” and 30.8% (*n* = 61) were “generally happy” about their pregnancy.

Approximately one-third of the participants had a bachelor’s degree or higher (*n* = 75, 37.9%) or had completed some college (*n* = 60, 30.3%) or high school (*n* = 59, 29.8%). Nearly two-thirds were employed (*n* = 126, 63.6%). Of those who were employed, 74.6% (*n* = 93) had paid maternity leave, whereas 1.6% (*n* = 2) did not.

Nearly three-fourths of the participants gave birth vaginally (*n* = 141, 71.2%). The mean birthweight of their newborn was 3,021.86 (*SD* = 358.18, range = 1,968–3,940) grams, and the mean gestational age at birth was 38.16 (267 days, *SD* = 1.37, range = 32–40) weeks. Nineteen babies (9.6%) were born prematurely, and 11 babies (5.6%) were low birthweight.

The majority of the participants breastfed their baby (*n* = 186, 93.7%). A large majority reported that their baby woke up several times during the night (*n* = 177, 89.7%). In regard to their baby’s sleep pattern, 44.4% (*n* = 88) thought it was a small problem, and 36.5% (*n* = 72) thought that, although serious, the problem was manageable. The majority of the participants had someone taking care of them (*n* = 194, 98.4%) and their baby (*n* = 161, 81.7%) during their first month postpartum.

### Differences in quality of life by demographic variables

As shown in Table 1, at T1, unemployed participants scored lower on mental, social, and general health than did employed participants. Participants who were happy about their pregnancy perceived having better mental and general health than did participants who were not happy about their pregnancy. Participants who had a higher educational level had better mental health than did their counterparts. Primiparas had a higher score on general health. However, age was not related to any dimension of QoL (*r* ranged from -.13 to .09). At postpartum (T4), employed participants were generally, mentally, and socially healthier.

**Table 1:** Differences on quality of life by demographic variables at T1 and T4

		**Physical health**			**Mental health**			**Social health**			**General health**		
	***n***	***M*±*SD***	***Z/X***^***2***^	***p***	***M*±*SD***	***Z/X***^***2***^	***p***	***M*±*SD***	***Z/X***^***2***^	***p***	***M*±*SD***	***Z/X***^***2***^	***p***
**T1**													
**Parity**			-.77	.44	−1.80		.07	−1.62		.10	−2.06		.04
Primipara	111	60.90±15.29			71.26±16.24			67.30±16.29			66.49±11.70		
Multipara	87	57.82±20.37			66.09±19.96			62.53±17.73			62.15±14.77		
**Employment**			-.51	.61	−4.05		<.001	−3.06		.002	−3.39		.001
Employed	126	60.16±17.43			72.78±18.09			67.94±16.94			66.96±13.11		
Unemployed	72	58.47±18.28			62.36±16.23			60.42±16.31			60.42±12.61		
**Education**			-.24	.81	−2.10		.04	−1.52		.13	−1.15		.25
Lower than college	63	59.84±20.12			64.76±19.25			62.22±16.50			62.28±14.87		
College or higher	135	59.41±16.56			70.96±17.27			66.59±17.20			65.65±12.38		
**Happy or unhappy about the pregnancy**		1.07		.59	8.70		.01	3.59		.17	6.94		.03
Happy	160	60.06±17.43			70.25±17.55			66.06±17.20			65.46±12.77		
Unhappy	13	54.62±15.06			56.92±10.32			57.69±14.23			56.41±8.97		
Uncertain	25	58.80±20.88			67.20±22.46			63.60±17.05			63.20±16.82		
**T4**													
**Parity**			-.71	.48	-.24		.81	−1.09		.28	-.09		.93
Primipara	111	59.55±19.42			69.19±20.85			61.62±15.46			66.79±15.54		
Multipara	87	70.57±20.65			70.11±21.16			58.51±17.56			66.40±16.27		
**Employment**		−1.51		.13	−3.21		.001	−2.71		.01	−3.02		.003
Employed	116	71.81±19.32			73.45±20.26			63.19±14.12			69.48±14.94		
Unemployed	82	67.44±20.60			64.15±20.78			56.10±18.58			62.56±16.24		
**Being taken care of**			-.17	.87	-.68		.50	-.18		.86	-.21		.83
Yes	192	70.10±19.66			69.43±20.70			60.31±16.37			66.61±15.63		
No	6	66.67±29.44			75.00±29.50			58.33±20.41			66.67±23.29		
**Baby sitter**		−1.66		.10	−1.59		.11	−1.77		.08	−1.84		.07
Yes	155	71.23±19.72			70.90±20.24			61.35±16.00			67.83±15.22		
No	43	65.58±20.27			64.88±22.93			56.28±17.60			62.25±17.35		
**Baby’s gender**			-.67	.51	−1.73		.08	-.34		.73	−1.32		.19
Male	106	70.94±20.07			71.98±20.54			60.75±15.16			67.89±15.65		
Female	91	69.23±19.68			67.14±21.10			59.67±17.98			65.35±15.95		
**Baby’s sleep patterns**		3.70		.16	1.49		.48	2.81		.25	3.39		.18
Serious problem	15	60.00±25.07			64.67±24.75			56.67±13.45			60.44±18.38		
Manageable	73	69.04±18.12			67.81±21.42			57.67±15.14			64.84±14.98		
Small problem	89	71.46±19.57			71.24±19.70			61.24±16.91			67.98±15.56		
Not a problem	21	74.29±22.26			72.38±21.89			67.62±18.95			71.43±16.88		
**Maternity leave**			.20	.91	2.24		.33	4.00		.14	1.55		.46
Paid leave	96	73.33±18.33			74.58±19.41			64.79±13.69			70.90±13.83		
No-pay leave	26	69.62±22.36			69.23±22.96			56.92±18.28			65.26±19.05		
No leave/not employed	76	65.92±20.47			63.42±20.69			55.66±17.61			61.67±15.68		
**Birth method**		−1.63		.10	-.19		.85	-.30		.77	-.68		.50
Vaginal delivery	143	60.98±17.21			69.51±17.78			65.38±17.11			65.29±12.61		
Cesarean section	54	56.48±18.14			68.15±18.84			64.44±17.12			63.02±14.81		

T1 = 25–29 gestational weeks; T4 = 4–6 weeks postpartum.

### Level of quality of life by time

Quality of life was analyzed through participants’ scores in general health, physical health, mental health, and social health by using the GEE method and controlling for demographic variables that showed differences on the QoL variables. As shown in Table 2, participants did not have very high scores in any dimension of QoL at all four data collection time periods. Compared to their perceptions of physical health at T1 (*M ± SD* = 59.55 ± 17.72), participants perceived that they had poorer physical health at T2 (*M ± SD* = 55.8 4 ± 18.49, *p* = .001) and T3 (*M ± SD* = 50.77 ± 18.58, *p* < .001); however, they perceived having better physical health at T4 (*M ± SD* = 70.00 ± 19.92, *p* < .001). Participants’ mental health did not change from T1 to T4 after controlling for demographic variables (*M ± SD* = 68.99 ± 18.11, 70.41 ± 18.48, 69.28 ± 20.89, and 69.60 ± 20.94 for T1, T2, T3, and T4 respectively; *p* > .05). Participants’ scores for social health were lower at postpartum than at T1 (*M ± SD* = 65.20 ± 17.06 and 60.25 ± 16.45 for T1 and T4 respectively; *p* < .001). Compared to their general health at T1, participants had poorer general health at T3 after controlling for employment and happiness about pregnancy.

**Table 2:** Comparison of dimensions of quality of life by data collection time period

	**Range**	***M*±*SD***	***B***	***Wald X***^***2***^	***p***	**95% CI**
**Physical health**						
T1 (referent)	0–100	59.55±17.72	-	-	-	-
T2	0-100	55.84±18.49	−3.66	11.53	.001	−5.77, -1.55
T3	0-90	50.77±18.58	−8.82	48.01	<.001	−11.32, -6.33
T4	10-100	70.00±19.92	10.46	41.33	<.001	7.27, 13.65
**Mental health**						
T1 (referent)	10–100	68.99±18.11	-	-	-	-
T2	20–100	70.41±18.48	1.52	1.63	.20	-.81, 3.85
T3	20–100	69.28±20.89	.30	.06	.81	−2.04, 2.63
T4	20–100	69.60±20.94	.61	.15	.70	−2.47, 3.69
Unhappy (referent)	50-80	56.92±10.32	-	-	-	-
Happy	20-100	70.25±17.55	3.61	1.05	.31	−3.29, 10.51
Uncertain	10-100	67.20±22.46	-.60	.02	.90	−9.64, 8.44
Lower than college (referent)	10-100	64.76±19.25	-	-	-	-
Equal to or higher than college	20-100	70.96±17.27	.10	.001	.97	−4.90, 5.09
Unemployed	20-100	62.36±16.23	-	-	-	-
Employed	10-100	72.78±18.09	8.26	11.33	.001	2.46, 3.45
**Social health**						
T1 (referent)	20–100	65.20±17.06	-	-	-	-
T2	10–100	64.87±18.70	-.27	.07	.80	−2.35, 1.80

Demographic variables that showed differences on the quality of life variables were controlled for in these analyses. T1 = 25–29 gestational weeks; T2 = 30–34 gestational weeks; T3= over 34 gestational weeks; T4 = 4–6 weeks postpartum.

**Figure 1:**
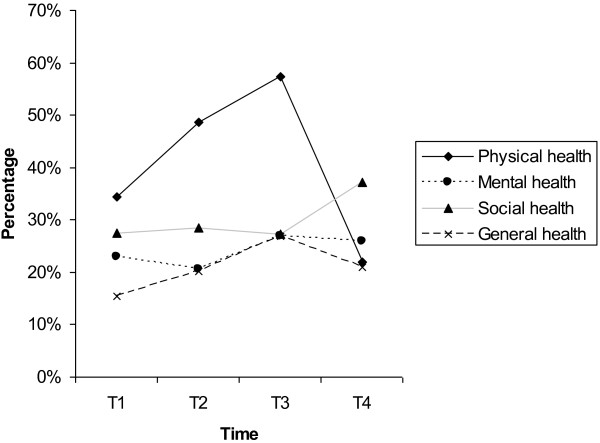
Rate of perceived poor/fair health from pregnancy to postpartum.

Using a score of 50 as the cutoff point (DUKE scale score ranges from 0 to 100) for poor/fair health or good/excellent health, we found that the rate of poor/fair physical health increased from 34.5% to 48.7% and to 57.4% at T1, T2, and T3, respectively, and decreased to 22.0% at T4. The rates of poor/fair mental health remained stable throughout pregnancy and postpartum (23%, 20.6%, 26.9%, and 26% at T1, T2, T3, and T4 respectively). Different from the trend for physical health, rates of poor/fair social health remained stable throughout pregnancy (27.4%, 28.4%, and 27.2% at T1, T2, and T3, respectively) and increased at postpartum (37.1%). For general health, the rate of poor/fair general health increased from 15.5% at T1 to 20.1% at T2 and 26.9% at T3, and decreased to 21.0% at T4. Figure 1 shows the trends for perceived poor/fair health from pregnancy to postpartum.

### Correlations between dimensions of quality of life

As shown in Table 3, the majority of general, physical, mental, and social health data at all collection time periods were significantly and positively intercorrelated except for T1 physical health and T4 social health (*r* = .11, *p* = .13). If one uses Cohen’s criteria for statistical power, where correlation coefficients lower than .30 denote a medium relationship and .10 a weak relationship [28], many correlations between prenatal variables and postpartum variables (except for prenatal and postpartum general health) were weak. In contrast, the majority of correlations among prenatal variables were medium or strong.

**Table 3:** Correlations between measured variables at different data collection time periods

	**1**	**2**	**3**	**4**	**5**	**6**	**7**	**8**	**9**	**10**	**11**	**12**	**13**	**14**	**15**
1. T1 physical health	1														
2. T1 mental health	.39^**^	1													
3. T1 social health	.21^*^	.44^**^	1												
4. T1 general health	.71^***^	.82^***^	.72^***^	1											
5. T2 physical health	.65^***^	.29^*^	.34^**^	.57^***^	1										
6. T2 mental health	.40^**^	.58^***^	.45^**^	.64^***^	.37^**^	1									
7. T2 social health	.26^*^	.49^**^	.66^***^	.62^***^	.27^*^	.49^**^	1								
8. T2 general health	.57^***^	.59^***^	.63^***^	.80^***^	.72^***^	.81^***^	.77^***^	1							
9. T3 physical health	.52^***^	.36^**^	.29^*^	.52^***^	.68^***^	.37^**^	.28^*^	.58^***^	1						
10. T3 mental health	.34^**^	.64^***^	.46^**^	.64^***^	.40^**^	.71^***^	.50^***^	.71^***^	.44^**^	1					
11. T3 social health	.23^*^	.41^**^	.62^***^	.56^***^	.32^**^	.51^***^	.63^***^	.64^***^	.27^*^	.57^***^	1				
12. T3 general health	.46^**^	.60^***^	.58^***^	.73^***^	.59^***^	.68^***^	.60^***^	.82^***^	.72^***^	.87^***^	.77^***^	1			
13. T4 physical health	.26^*^	.20^*^	.22^*^	.30^**^	.43^**^	.23^*^	.23^*^	.39^**^	.36^**^	.27^*^	.22^*^	.36^**^	1		
14. T4 mental health	.18^*^	.35^**^	.29^*^	.36^**^	.34^**^	.41^**^	.32^**^	.47^**^	.27^*^	.41^**^	.29^*^	.42^**^	.58^***^	1	
15. T4 social health	.11	.26^*^	.44^**^	.36^**^	.23^*^	.27^*^	.50^***^	.44^**^	.21^*^	.24^*^	.42^**^	.36^**^	.40^**^	.57^***^	1
16. T4 general health	.23^*^	.33^**^	.37^**^	.41^**^	.41^**^	.37^**^	.41^**^	.52^***^	.35^**^	.38^**^	.37^**^	.46^**^	.82^***^	.89^***^	.77^***^

All correlations were significant at *p* < .05 except for correlation between T1 physical health and T4 social health (*p*=.13). ^*^weak relationship, ^**^medium relationship, ^***^strong relationship based on Cohen’s criteria of power. T1 = 25–29 gestational weeks; T2 = 30–34 gestational weeks; T3= over 34 gestational weeks; T4 = 4–6 weeks postpartum.

### Prediction of newborn preterm birth and low birthweight

The dimensions of QoL were tested for their predictive ability for preterm birth and infant low birthweight using two hierarchical logistic regressions. Before the analysis, demographic variables were tested for their relationships with preterm birth and low birthweight by the Mann–Whitney *U* test or Kruskal-Wallis test. Results showed that only parity (*Z* = −3.73, *p* < .001) was related to preterm birth; none of the other demographic variables showed a significant relationship. Therefore, in the regression analysis for preterm birth, parity was controlled for by entering in the first set, while variables measured at T1 were in the second set, variables at T2 in the third set, and variables at T3 in the fourth set. For low birthweight analysis, neither parity nor any other variable was controlled for because no demographic variable was related to low birthweight. The results showed that physical health at T2 and all dimensions of QoL at T3 could predict preterm birth (Table 4). In other words, after controlling for all other variables in the analysis, pregnant women who had a higher score on T2 physical health had a higher risk of giving birth prematurely by 1.09 times. After controlling for all other variables in the analysis, those who had a lower score on T3 physical health and social health had a lower risk of giving birth prematurely by 5% and 9%, respectively, whereas those who had a higher score on T3 mental health had a higher risk of giving birth prematurely by 1.07 times.

**Table 4:** predictive ability of quality of life for preterm birth and low birthweight

	***B***	**S.E.**	**Wald**	***p***	**Exp(B)**
**Preterm birth**					
Constant	−2.26	1.52	2.23	.14	.10
Parity	−2.51	.84	8.91	.003	.08
T1 physical health	-.004	.03	.02	.89	1.00
T1 mental health	-.04	.02	3.00	.08	.96
T1 social health	.05	.03	1.95	.16	1.05
T2 physical health	.09	.04	6.18	.01	1.09
T2 mental health	-.03	.03	1.33	.25	.97
T2 social health	.03	.03	1.18	.28	1.03
T3 physical health	-.06	.03	5.01	.03	.95
T3 mental health	.06	.03	5.58	.02	1.07
T3 social health	-.10	.03	10.03	.002	.91
**Low birthweight**					
Constant	−2.81	2.08	1.83	.18	.06
T1 physical health	-.01	.04	.14	.71	.99
T1 mental health	-.09	.03	7.50	.01	.92
T1 social health	.01	.04	.02	.89	1.01
T2 physical health	.02	.04	.16	.69	1.02
T2 mental health	-.03	.03	1.25	.26	.97
T2 social health	.09	.03	6.80	.01	1.09
T3 physical health	-.01	.03	.10	.75	.99
T3 mental health	.06	.03	2.98	.08	1.06
T3 social health	-.04	.04	.86	.36	.96

The finding that higher T3 mental health score (better mental health) could predict preterm birth was inconsistent with other study findings [6], we therefore conducted a further analysis and found that 40% (n = 2 out of 5) of pregnant women who had a low mental health score at T3 (scored 0–25) gave birth prematurely. Additionally, the rate of women who gave birth prematurely was 4.2% (n = 2 out of 48) for those who scored median low (scored 25.1–50), 6.8% (n = 4 out of 59) for those who scored median high (scored 50.1–75), and 10.6% (n = 9 out of 83) for those who scored high (scored 75.1–100), and the rate was significantly different by groups (*X*^*2*^ = 9.86, *p* = .02). These results showed that, in fact, the pregnant women who had a very low score on mental health (score lower than 25) had higher risk of giving birth prematurely than women had higher scores (score higher than 25).

For low birthweight, only mental health at T1 and social health at T2 were included in the regression model (Table 4). In other words, after controlling for other variables in the analysis, pregnant women who had a higher score on T1 mental health had a lower risk of giving birth to a low birthweight infant by 8%. while those who had a higher score on T2 social health had a higher risk of giving birth to a low birthweight infant by 1.09 times.

## Discussion

### Maternal physical health

Throughout their pregnancy and early postpartum period, the women in this study did not perceive having a high level of QoL, which included physical, mental, social, and general health. In fact, the women’s perceived level of physical health decreased as their pregnancy progressed. This finding is consistent with results from previous studies in other countries, which suggests that pregnant women, regardless of their ethnicity and culture, perceive having poorer physical health later in their pregnancy. In a study conducted in the United States, Hass et al. found that pregnant women’s physical function and vitality, which can be considered factors related to physical health, declined during pregnancy, especially at late pregnancy [18]. Lau and Yin’s survey of the QoL of Chinese pregnant women in their second trimester found that, overall, the pregnant women had a low score on physical health when measured with the SF-12, a scale for health-related QoL [10]. In Sweden, Schytt and Hildingsson found that 20.4% of the pregnant women in their study perceived having poor/fair physical health at mid-pregnancy, and the rate increased to 36.9% in late pregnancy [17]. Our study revealed similar findings, specifically that the rate of poor/fair physical health increased from T1 to T3. The increase in pregnancy-related physical symptoms, especially symptoms that occur or become severe at late pregnancy, may explain these changes in the level of women’s perceived physical health throughout their pregnancy. For example, in Rodriguez et al.’s longitudinal study, 8% of women pregnant at 20 weeks frequently experienced difficulty sleeping, while the rate increased to 17% at 28 weeks and 25% at 32–36 weeks of gestation [29].

At one-month postpartum, the women in our study perceived having better physical health compared to their physical health during pregnancy. We found the rate of poor/fair physical health at T4 dramatically decreased from the rate at T3. This result is similar to that of Schytt and Hildingsson, who found that, at two-months postpartum, the rate of poor/fair physical health of the women in their study decreased to 19.9% from the 36.9% rate at late pregnancy [17]. Perhaps the mothers perceived having better physical health after childbirth because they no longer had the burden of pregnancy-related physical health challenges.

### Maternal mental health

The women in our study had a median high score on mental health (mean scores ranged from 69.25 to 70.60), and the score did not change significantly throughout the women’s pregnancy or into their postpartum period. We also found that the rate of perceived poor/fair mental health of women remained similar from pregnancy to postpartum. The poor mental health status of the women in our study throughout their pregnancy and into their early postpartum period is similar to what was found in research from other countries. Schytt and Hildingsson found that 14.3% to 23.9% of pregnant women perceived having poor mental health from mid-pregnancy to postpartum; the women also perceived having poorer mental health than that of the general population [17]. Gartland et al. also found that Australian pregnant women perceived having poorer mental health at 30–32 weeks of gestation than that of the general population [9].

Among the studies on mental health issues in women throughout pregnancy and postpartum, depression or depressive symptoms have been the main focus, and postpartum depression has been examined more often than has prenatal depression. In a community-based study conducted in Canada, researchers found that 14.1%, 10.4%, and 8.1% of women in early pregnancy, late pregnancy, and postpartum, respectively, experienced depressive symptoms [30]. In a study conducted in China, the rate of depression and/or anxiety was 15% among women at all stages of pregnancy [31]. The high rate of pregnant and postpartum women’s experiencing poor mental health, as evidenced in our study and in previous studies, and the relationship between poor mental health and preterm birth and low birthweight, as found in our study, highlight the need for more research that, in addition to a focus on depression or anxiety, focuses on other negative affects as well.

### Maternal social health

The pregnant women in our study did not score high on social health, and their perceived social health decreased in the postpartum period compared to earlier in their pregnancy. Previous studies found that pregnant women perceived having poorer social function than did non-pregnant women or people in general [9,16]. Although our study did not survey non-pregnant women, the low social health score of the participants might be consistent with results from those earlier studies. An explanation for the decreased perception of social health among the Taiwanese participants in our study may be the traditional Chinese cultural practice of “doing the month,” which confines the new mother at home for a month while someone takes care of her [32,33]. It might also explain why study participants who were employed perceived having better social health, both during pregnancy and after childbirth, than did unemployed participants.

### Maternal general health

In our study, the pregnant women’s perceived level of general health decreased at late pregnancy. Using a score of 50 as the cutoff point (DUKE scale score ranges from 0 to 100) for poor/fair general health or good/excellent general health, we determined that the rate of poor/fair general health was 15.5%, 20.1%, 26.9%, and 21.0% at T1, T2, T3, and T4, respectively. Although the use of different measuring tools makes it difficult to compare our results with results from other studies, the rate of poor/fair health experienced by the Chinese/Taiwanese pregnant women in our current study was higher than the rate of American pregnant women in Hass et al.’s study. The perceived general health status of the participants in Hass et al.’s study did not change significantly over the course of pregnancy and included a rate of less than 15% for poor/fair health [18]. The reason for the difference in rates between our study and that of Hass et al. may be that people from different cultures and ethnicities perceive health differently.

In our study, women who were employed and happy about their pregnancy perceived having better general health than did women who were unemployed and unhappy about their pregnancy. This finding suggests that encouraging women to view the positive aspects of their pregnancy may help promote their general health status.

### Predictors of newborn preterm birth and low birthweight

The results from our study suggest that all dimensions of maternal QoL at late pregnancy can predict preterm birth and that maternal physical health at mid-pregnancy can predict infant low birthweight. Although no research has reported on the relationships of maternal QoL with preterm birth and with low birthweight, some studies have found that previous or present pregnancy complications, multiple fetuses, low psychosocial health, more negative affects, a lower level of perceived social support, and several social health issues are related to premature birth or low birthweight [4-6,24]. Similarly, our study found that poor physical health and poor social health at late pregnancy can predict preterm birth and that poor mental health at early pregnancy can predict low birthweight.

One finding in our study that was inconsistent with other studies is that better, rather than poorer, mental health at late pregnancy can predict preterm birth. We also found that, in fact, the pregnant women who had a very low score on mental health had a higher risk of giving birth prematurely than did women who had higher scores. We therefore suggest that assessing maternal mental health actively and paying more attention on pregnant women with very poor mental health status.

### Study limitations

The strengths of this study are its longitudinal design and the participants’ low attrition rate. However, the generalizability of the study’s findings is limited due to the use of convenience sampling in just one hospital located in Taiwan. To increase the generalizability of our findings, we recommend that future longitudinal investigations involve other sampling methods of women throughout their pregnancy and postpartum periods as well as participants from more hospitals.

## Conclusions

The findings from our study fill a gap in the literature and provide an important longitudinal view of maternal QoL dimensions during pregnancy and the postpartum period as well as their influences on maternal health and birth outcomes. Increasing the understanding of these factors and implementing appropriate interventions can help reduce not only maternal morbidity and mortality but also the incidence of preterm birth and low birthweight infants.

The results of our study suggest that all the dimensions of maternal QoL are intercorrelated throughout the continuum of pregnancy and the postpartum period. The pregnant women in our study did not perceive having a high level of QoL. Compared to their levels of QoL early in pregnancy, the women experienced a decrease in their level of physical and general health later and throughout their pregnancy. Moreover, the maternal demographic variables of parity (primiparas were generally healthier), employment (employed women were mentally, socially, and generally healthier), educational level (women with higher education level were mentally healthier), and happiness about pregnancy (women who were happy about pregnancy were mentally and generally healthier) were related to prenatal QoL, whereas employment (employed women were mentally, socially, and generally healthier) was related to postpartum maternal QoL. After childbirth, the mothers perceived having better physical health, they had poorer social health. We also found that, among our study’s sample, poor maternal physical and social health at late pregnancy could predict preterm birth and that low maternal mental health scores at earlier pregnancy tended to predict infants born with a low birthweight.

Based on our study’s findings, we suggest that additional research is needed to provide a more thorough understanding of all the dimensions of QoL among women throughout pregnancy and the postpartum period and, especially, of the significant effect of these factors on maternal health and birth outcomes. An emphasis on the positive aspects of pregnancy may increase maternal QoL. We also recommend devoting more attention to developing preventive interventions that specifically address pregnant women’s QoL and to implementing them early in pregnancy to prevent pregnant women’s low levels of perceived QoL and to promote high levels of QoL at late pregnancy and beyond. These interventions may help prevent preterm birth and low birthweight infants. In our study, we used the Chinese version of the DUKE provided by the scale developer and tested the reliability of the scale only when used with Chinese/Taiwanese in Taiwan. We also suggest that, if future studies use instruments developed in a language other than the language used by the study population, the researchers need to follow the process for translation and validation [34].

## Competing interest

The authors declare that they have no competing interests.

## Authors’ contributions

PW participated in the study design, collected the data, and finalized the manuscript. SRL designed the study and drafted the manuscript. CYC participated in the study design, collected the data, analyzed the data, and finalized the manuscript. All authors read and approved the final manuscript.

## Pre-publication history

The pre-publication history for this paper can be accessed here:

http://www.biomedcentral.com/1471-2393/13/124/prepub
